# A Functional *TNFAIP2* 3'-UTR rs8126 Genetic Polymorphism Contributes to Risk of Esophageal Squamous Cell Carcinoma

**DOI:** 10.1371/journal.pone.0109318

**Published:** 2014-11-10

**Authors:** Jian Zhang, Hongchen Yu, Yi Zhang, Xiaoshi Zhang, Guixin Zheng, Yang Gao, Chuanxin Wang, Liqing Zhou

**Affiliations:** 1 Department of Clinical Laboratory, Qilu Hospital of Shandong University, Jinan, Shandong Province, China; 2 Qingdao Qilu Hospital, Shandong University, Qingdao, Shandong Province, China; 3 Department of Radiation Oncology, Huaian No. 2 Hospital, Huaian, Jiangsu Province, China; National Cancer Institute, National Institutes of Health, United States of America

## Abstract

**Background:**

Accumulated evidences demonstrated that single nucleotide polymorphisms (SNPs) in mRNA 3'-untranslated region (3'-UTR) may impact microRNAs (miRNAs)-mediated expression regulation of oncogenes and tumor suppressors. There is a *TNFAIP2* 3'-UTR rs8126 T>C genetic variant which has been proved to be associated with head and neck cancer susceptibility. This SNP could disturb binding of miR-184 with *TNFAIP2* mRNA and influence *TNFAIP2* regulation. However, it is still unclear how this polymorphism is involved in development of esophageal squamous cell carcinoma (ESCC). Therefore, we hypothesized that the functional *TNFAIP2* rs8126 SNP may affect *TNFAIP2* expression and, thus, ESCC risk.

**Methods:**

We investigated the association between the *TNFAIP2* rs8126 variant and ESCC risk as well as the functional relevance on *TNFAIP2* expression *in vivo*. Genotypes were determined in a case-control set consisted of 588 ESCC patients and 600 controls. The allele-specific regulation on *TNFAIP2* expression by the rs8126 SNP was examined in normal and cancerous tissue specimens of esophagus.

**Results:**

We found that individuals carrying the rs8126 CC or CT genotype had an OR of 1.89 (95%CI  = 1.23–2.85, *P = *0.003) or 1.38 (95%CI  = 1.05–1.73, *P = *0.017) for developing ESCC in Chinese compared with individual carrying the TT genotype. Carriers of the rs8126 CC and CT genotypes had significantly lower *TNFAIP2* mRNA levels than those with the TT genotypes in normal esophagus tissues (*P<*0.05).

**Conclusions:**

Our data demonstrate that functional *TNFAIP2* rs8126 genetic variant is a ESCC susceptibility SNP. These results support the hypothesis that genetic variants interrupting miRNA-mediated gene regulation might be important genetic modifiers of cancer risk.

## Introduction

As one of the most common and fatal malignancies worldwide, esophageal squamous cell carcinoma (ESCC) shows a relatively high morbidity in Eastern Asian and Eastern African compared to Western countries [1]. The ESCC etiology is still not completely clear, though tobacco smoking, heavy alcohol drinking, micronutrient deficiency as well as dietary carcinogen exposure have been proven to be major environmental causes [2], [3]. In Chinese, low consumption of vegetables and fruits, limited Vitamin C intake, and high temperature of meals and drinks were strong risk indicators of ESCC in Chinese populations. In addition, the strength of tea and overall tea consumption were independent determinants of the ESCC risk [3]. However, there were only a part of exposed individuals developing ESCC, indicating the involvement of genetic components in development of this lethal disease [2]–[9].

TNFAIP2 (tumor necrosis factor, alpha-induced protein 2) is also known as the primary response gene B94 protein, which is one of the SEC6 members. Originally, TNFAIP2 was identified as a TNF-alpha-induced protein in human endothelial cells [10]. As a target gene of retinoic acid in acute pro-myelocytic leukemia and other cancers, TNFAIP2 plays an important role in controlling of apoptosis [11]–[13]. Higher expressed TNFAIP2 in nasopharyngeal carcinoma tumor specimens was found compared to adjacent normal tissues. The elevated TNFAIP2 expression was significantly associated with shorter distant metastasis-free survival among patients with nasopharyngeal carcinoma [14]. Interestingly, Liu et al identified a functional rs8126 T>C single nucleotide polymorphisms (SNP) within the miR-184 seed binding sequence in the 3'-untranslated region (3'-UTR) of *TNFAIP2* mRNA. In detail, the rs8126 variant C allele led to significantly lower luciferase activity and expression of TNFAIP2 mRNA, compared with the T allele [15]. This functional genetic variant is also significantly associated with susceptibility of head and neck squamous cell carcinoma (HNSCC) as well as gastric cancer [15], [16]. In HNSCC, compared with the rs8126 TT genotype, the variant C allele were associated with increased cancer risk in an allele dose-response manner (adjusted odds ratio [OR]  = 1.48, 95% confidence interval [CI]  = 1.06–2.05 for CC, respectively; *P*_trend_  = 0.009). Similarly, the rs8126 CC genotype was associated with a significantly elevated risk of gastric cancer (adjusted OR = 2.00, 95% CI = 1.09–3.64, *P* = 0.024), compared with the combined rs8126 TT and TC genotypes, particularly in current drinkers.

However, it is still unclear whether this functional polymorphism is involved in ESCC development. Based on the aforementioned findings, we hypothesized that the *TNFAIP2* rs8126 genetic polymorphism may contribute to ESCC susceptibility via influencing TNFAIP2 expression in esophagus cells. To test this hypothesis, we conducted a large case-control study of ESCC. To reveal the biological function of this SNP, we detected the impact of its different genotypes on *TNFAIP2* mRNA expression in both normal and malignant esophagus tissues.

## Materials and Methods

### The case-control set

A total of 588 ESCC cases from Huaian No. 2 Hospital (Huaian, Jiangsu Province, China) and sex- and age-matched 600 controls were included in this study. Patients were consecutively recruited between January 2009 and February 2012 at Huaian No. 2 Hospital. All cases are incident ones during enrollment of the current case-control study. Cancer-free controls were chosen from 3600 individuals of a community cancer-screening program for early cancer detection. The program was conducted in Huaian city during the same time period as the patients were recruited. The diagnosis of all patients was histologically confirmed. Subjects who smoked one cigarette per day for more than one year were considered as smokers. Individuals were considered as alcohol drinkers, if they drank at least once every week. Eighteen pairs of ESCC tissues and esophagus normal tissues adjacent to the tumors were obtained from surgically removed specimens of patients in Huaian No. 2 Hospital. The normal tissues sampled at least 2cm away from the margin of the tumor. All subjects were ethnic Han Chinese. At recruitment, the informed consent was obtained from each subject. All participants have provided their written informed consents to participate in this study. This study was approved by the institutional Review Board of Huaian No. 2 Hospital.

### Genotyping

The rs8126 T>C polymorphism was genotyped through the PCR-restriction fragment length polymorphism (RFLP) method as described previously [15]. In brief, the primers used for amplifying DNA segments with the SNP site (the mismatch base is underlined) were 5′- GGGGCCGGCTCTCTTGGGCC-3′ and 5′-CACACGTACAAAGACCTTGGGCATCC-3′. The 25 µL PCR reaction mixture consisted of 100 ng of DNA, 0.1 mmol/L of each primer, 0.2 mmol/L of deoxynucleoside triphosphate, 1.0 U of Taq DNA polymerase (New England Biolabs, NEB), 1× reaction buffer and 1.5 mmol/L MgCl_2_. The PCR profile contains an initial melting step of 2 minutes at 94°C, followed by 35 cycles of 30 seconds at 94°C, 30 seconds at 55°C, 30 seconds at 72°C, and a final elongation step of 10 minutes at 72°C. Restriction enzyme *Apa*I (NEB) was utilized to distinguish the rs8126 genotypes. A 10% random sample was reciprocally examined by different persons, and the reproducibility was 100%.

### Real-time analyses

Total RNA was extracted from cancerous and normal esophagus tissue samples using TRIzol Reagent following RNase-Free DNase treatment (Invitrogen). RNA was examined in 1% agrose gel which was stained with EB to examine the integrity of each tissue RNA sample before qRT-PCR. The quantity was detected by Nanodrop and 100 ng RNA was used in the RT reaction with TaqMan Reverse Transcription Reagents (N8080234). TaqMan real-time qPCR method with the master mix reagent (Applied Biosystems, ABI) was used to examine mRNA levels in cancerous and normal esophagus tissues according to the manufacturer's instructions. The expression values of *TNFAIP2* mRNA or *β-actin* mRNA distributed normally. Relative gene expression quantization for *TNFAIP2* (ABI catalog ID: Hs00969310_m1) was examined with the ABI 7500 real-time PCR system in triplicates using *β-actin* as the endogenous control (ABI catalog ID: 4333762T). The expression of individual *TNFAIP2* mRNA measurements was calculated relative to expression of *β-actin* [17].

### Statistics

The differences in demographic variables and genotype distributions of *TNFAIP2* rs8126 T>C polymorphism between ESCC patients and controls were calculated by Pearson's χ^2^ test. Associations between *TNFAIP2* rs8126 genotypes and ESCC risk were estimated by OR and their 95% CIs computed using unconditional logistic regression model. All ORs and 95% CIs were adjusted for age, sex, drinking and smoking status, where it was appropriate. All statistical tests were two-sided. A *P* value of less than 0.05 was used as the criterion of statistical significance. All calculations were performed with SPSS software package (Version 16.0, SPSS Inc., Chicago, IL).

## Results

### Association between *TNFAIP2* rs8126 SNP and ESCC risk

No statistically significant differences were found between ESCC cases and healthy controls in terms of median age and sex distributions (*P*>0.05), indicating that the frequency matching was adequate (Table 1). However, more smokers and alcohol drinker were observed among patients compared with control subjects.

**Table 1:** Distribution of selected characteristics among ESCC cases and controls.

Variable	Cases	Controls	*P* 1
	No. (%)	No. (%)	
	588	600	
Age (year)^2^			0.725
≤59	288(49.0)	300(50.0)	
>59	300(51.0)	300(50.0)	
Sex			0.678
Female	175(29.8)	172(28.7)	
Male	413(70.2)	428(71.3)	
Smoking status			<0.001
No	151(25.7)	397(66.2)	
Yes	437(74.3)	203(33.8)	
Drinking status			<0.001
No	254(43.2)	358 (59.7)	
Yes	334(56.8)	242(40.3)	

Abbreviation: ESCC, esophageal squamous cell carcinoma.

1 Two-sided χ^2^ test.

2 Median age of cases is 59 years.

Allele frequencies and genotype distributions of *TNFAIP2* rs8126 T>C SNP in ESCC cases and controls are shown in Table 2. The allele frequencies for rs8126 C were 0.323 in cases and 0.264 in controls. All observed genotype frequencies in both controls and cases conform to Hardy-Weinberg equilibrium. Distributions of the rs8126 genotypes were then compared among ESCC patients and controls. Frequencies of rs8126 TT, CT, and CC genotypes among cases differed significantly from those among controls (*P = *0.006), with the frequency of CC homozygote being significantly higher among patients than among controls (9.7% vs. 6.2%).

**Table 2:** Genotype frequencies of the *TNFAIP2* rs8126 polymorphism among ESCC cases and controls and their association with ESCC risk.

Genotypes	Cases No. (%)	Controls No. (%)	OR^1^ (95% CI)	*P* 1
rs8126				
	*n* = 588	*n* = 600		
TT	265(45.1)	320(53.3)	Reference	
CT	266(45.2)	243(40.5)	1.38(1.05–1.73)	0.017
CC	57(9.7)	37(6.2)	1.89(1.23–2.85)	0.003
C allele	0.323	0.264		
*P*_trend_			0.001	

Abbreviation: ESCC, esophageal squamous cell carcinoma; OR, odds ratio; CI, confidence interval.

1 Data were calculated by logistic regression with adjustment for age, sex, smoking and drinking status.

2 Test for trend of odds was two-sided and based on likelihood ratio test assuming an additive model.

Associations between genotypes of *TNFAIP2* rs8126 genetic polymorphism and ESCC risk were estimated using unconditional logistic regression analyses (Table 2). The rs8126 C allele was shown to be risk allele. Subjects with the rs8126 CC genotype had an OR of 1.89 (95% CI  = 1.23–2.85, *P* = 0.003) for developing ESCC in this population compared with individual having the TT genotype. Additionally, the rs8126 CT carriers also showed a 1.38-fold increased ESCC risk compared with those carrying the rs8126 TT genotype (95% CI  = 1.05–1.73, *P* = 0.017) (Table 2). Both ORs were adjusted for sex, age, alcohol drinking and smoking status. The stratified analyses demonstrated that the CC genotype was significantly associated with ESCC risk in individuals with different sex, age, drinking and smoking status. No gene-environment interactions existed between *TNFAIP2* rs8126 polymorphism and sex, age, alcohol drinking or smoking status.

### Functional relevance of rs8126 on *TNFAIP2* expression

**Figure 1:**
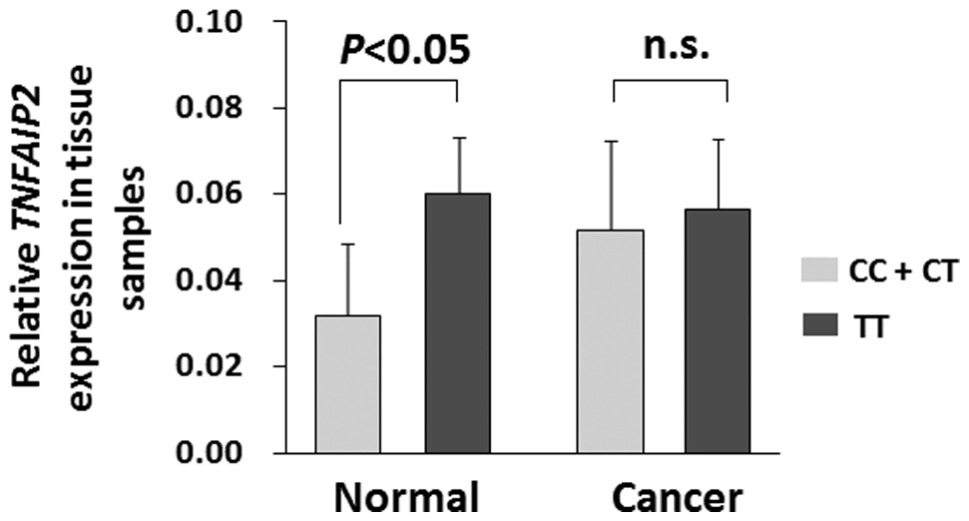
*TNFAIP2* mRNA expression (mean ±SD) in normal and cancerous esophagus tissues grouped by *TNFAIP2* rs8126 T>C genotypes. The individual *TNFAIP2* mRNA expression was calculated relative to expression of *β-actin* mRNA using the 2^−dCt^ method. In normal esophagus tissues, significantly lower *TNFAIP2* mRNA levels among subjects with the rs8126 CC and CT genotypes were observed compared with the TT genotypes (0.0319±0.0167 [*n = *8] vs. 0.0602±0.0132 [*n = *10], *P<*0.05). However, there were no statistically significant differences of *TNFAIP2* mRNA expression were found between CC, CT and TT genotypes in ESCC tissue specimens (CC and CT: 0.0517±0.0207 [*n = *8] vs. TT: 0.0566±0.0163 [*n = *10], *P>*0.05, n.s., not significant).

We investigated whether the ESCC susceptibility SNP rs8126 has an allele-specific impact on the *TNFAIP2* mRNA expression in both ESCC and normal esophagus tissues in the same population. As shown in Figure 1, we found that individuals with the rs8126 CC and CT genotypes had significantly lower *TNFAIP2* mRNA levels (mean ±SD) than those with the TT genotypes in normal esophagus tissues (0.0319±0.0167 [*n = *8] vs. 0.0602±0.0132 [*n = *10], *P* = 0.013). However, no statistically significant differences of *TNFAIP2* expression were observed between CC, CT and TT genotypes in ESCC tissue specimens (CC and CT: 0.0517±0.0207 [*n = *8] vs. TT: 0.0566±0.0163 [*n = *10], *P* = 0.172).

## Discussion

In the current study, we investigated the association between the functional *TNFAIP2* 3'-UTR rs8126 genetic variant and ESCC risk via a case-control approach. To the best of our knowledge, this is the first study to examine how the *TNFAIP2* rs8126 SNP is involved in ESCC tumorigenesis. We found that the rs8126 genetic variant was significantly associated with increased ESCC risk in a Chinese population. Intriguingly, this polymorphism has an allele-specific impact on *TNFAIP2* expression *in vivo*. Our data support the hypothesis that functional genetic variants at gene 3'-UTR region influence miRNA-mediated expression regulation of mRNA.

Genome-wide association studies (GWAS) highlighted the importance of SNPs in ESCC development [18]–[23]. However, the polymorphisms identified by GWAS can only explain a small part of ESCC genetic basis. Therefore, there are still many to question how the remaining ‘missing’ heritability could be explained. Thus, discovery of novel biologically functional ESCC susceptibility SNPs is potentially valuable to elucidate ESCC genetics thoroughly.

In a previous study, Liu et al firstly identified a functional rs8126 T>C SNP in HNSCC [15]. They demonstrated that the T-to-C change caused by the SNP within the miR-184 seed binding region of the 3'-UTR of *TNFAIP2* mRNA could significantly decrease expression of *TNFAIP2* mRNA *in vitro* and *in vivo* [15]. In line with this, we also found that there was much lower *TNFAIP2* expression in normal esophagus specimens among C allele carriers than those in T allele carriers. After genotyping the *TNFAIP2* rs8126 polymorphism in 1077 HNSCC patients and 1073 cancer-free controls in a non-Hispanic White population, Liu et al found that, compared with the rs8126 TT genotype, the variant C allele were associated with increased HNSCC risk in an allele dose-response manner (OR = 1.48, 95% CI = 1.06–2.05 for the CC genotype, respectively; *P*_trend_  = 0.009) [15]. In addition, Xu et al conducted a case-control study of 301 gastric cancer patients and 313 cancer-free controls frequency matched by age, sex and ethnicity. They found that the rs8126 CC genotype was associated with a significantly elevated risk of gastric cancer (OR = 2.00, 95% CI = 1.09–3.64, *P* = 0.024), compared with the combined rs8126 TT and TC genotypes, particularly in current drinkers [16]. Consistent with these results, we also observed statistically significant association between rs8126 CC or CT genotype and ESCC risk in a Chinese Han population. These data demonstrated that the *TNFAIP2* rs8126 polymorphism might be a common risk factor among different gastrointestinal malignancies.

There might be several limitations in the current case-control study. For instance, inherent selection bias may exist since it was a hospital-based study and the cases were from the hospital. Therefore, the findings of this study warrant to be validated in the future through a population-based prospective study. Liu et al presented the rs8126 as a functional variant that affects binding of miR-184 and presumably regulates expression of TNFAIP2 in head and neck cancer. However, functionality of this variant could not be generalized to other tissues without testing, even though the current analysis showed nominal association of the polymorphism with mRNA expression.

In summary, our results demonstrated that the functional *TNFAIP2* rs8126 polymorphism was associated with a significantly increased risk of ESCC in a Chinese population. These data support the hypothesis that genetic variants interrupting miRNA-mediated gene regulation might be important genetic modifiers of cancer risk.
